# Biomarkers for early diagnosis of malignant mesothelioma: Do we need another moonshot?

**DOI:** 10.18632/oncotarget.17910

**Published:** 2017-05-17

**Authors:** Sabrina Lagniau, Kevin Lamote, Jan P. van Meerbeeck, Karim Y. Vermaelen

**Affiliations:** ^1^ Tumor Immunology Laboratory, Department of Respiratory Medicine, Ghent University Hospital, 9000 Ghent, Belgium; ^2^ Department of Internal Medicine, Ghent University, 9000 Ghent, Belgium; ^3^ Thoracic Oncology/MOCA, Antwerp University Hospital, 2650 Edegem, Belgium

**Keywords:** mesothelioma, biomarkers

## Abstract

Early diagnosis of malignant pleural mesothelioma (MPM) is a challenge for clinicians. The disease is usually detected in an advanced stage which precludes curative treatment. We assume that only new and non-invasive biomarkers allowing earlier detection will result in better patient management and outcome. Many efforts have already been made to find suitable biomarkers in blood and pleural effusions, but have not yet resulted in a valid and reproducible diagnostic one. In this review, we will highlight the strengths and shortcomings of blood and fluid based biomarkers and highlight the potential of breath analysis as a non-invasive screening tool for MPM. This method seems very promising in the early detection of diverse malignancies, because exhaled breath contains valuable information on cell and tissue metabolism. Research that focuses on breath biomarkers in MPM is in its early days, but the few studies that have been performed show promising results. We believe a breathomics-based biomarker approach should be further explored to improve the follow-up and management of asbestos exposed individuals.

## INTRODUCTION

Malignant pleural mesothelioma (MPM) is a rare and aggressive cancer originating from the mesothelial cells of the pleura. MPM is mostly an occupational disease, affecting more men than women [1]. Although the causal link to asbestos exposure is well documented, and the latter's industrial use has been fully banned in Europe, asbestos is still being processed in large parts of the developing world [2]. Asbestos is a general name for naturally occurring mineral silicate fibers (e.g. serpentine and amphiboles), popular for industrial usage because of their high tensile strength and resistance to thermal and chemical degradation [3]. In 1960 already, Wagner *et al*. reported that asbestos had genotoxic and carcinogenic properties [4]. When asbestos fibers are inhaled in the lungs, they cause oxidative stress and chronic inflammation. Because of the high iron content of these fibers, Fenton-like reactions take place, resulting in the constant generation of reactive oxygen species (ROS). Furthermore, chronic inflammation is due to the prolonged phagocytic activity of macrophages engulfing the inhaled asbestos fibers [5]. This process generates both ROS and reactive nitrogen species (RNS), which both cause DNA damage, resulting in large-scale alterations in chromosomal loci harboring tumor-suppressor genes such as NF2 and BAP1 [6]. Hence, mesothelioma is the prototypical illustration of the genotoxic effects of protracted tissue inflammation, culminating in carcinogenesis with a long latency period after initial asbestos exposure.

There are 3 major histological subtypes of MPM [7]: epithelioid mesothelioma is the most common one and has the best prognosis, and sarcomatoid mesothelioma showing the worst. Biphasic mesothelioma has both epithelioid and sarcomatoid subtypes combined in various proportions.

The prognosis of MPM is poor due to its non-specific clinical manifestations, responsible for a diagnosis in an advanced stage. Currently, diagnostic procedures for MPM involve imaging tests and a biopsy. However, there is an unmet need of sensitive and non-invasive screening tools that allow an early detection of the disease, considered a precondition for the improvement of the presently low 5 year survival rate of less than 5% [8].

Therefore, biomarkers can be explored and can be useful within three aspects of the clinical management of MPM: early diagnosis (diagnostic biomarkers), prognosis (prognostic biomarkers) and prediction of treatment outcome (predictive biomarkers) [9]. This review will focus on diagnostic biomarkers. The required characteristics of this type of biomarkers depend on whether they will be used for diagnosis or screening. In case of using the biomarker for diagnosis, the specificity and positive predictive value (PPV) should be high enough in order to confirm the disease in a true positive population. In a screening setting however, the sensitivity and negative predictive value (NPV) of the biomarker are important for ruling out the disease in a true negative population.

This review will highlight the findings of current research efforts concerning the use of several biomarkers for early diagnosis of MPM, and focus on the potential of breath analysis within this scope.

### Diagnostic biomarkers in mesothelioma: An overview of current research

#### Methods

We searched for relevant studies concerning biomarkers in mesothelioma through MEDLINE (PubMed Database) and Web of Science using the following keywords and their combinations: “mesothelioma”, “biomarker”, “diagnosis”, “tumor marker”, “mesothelin”, “fibulin-3”, “osteopontin”, “megakaryocyte potentiating factor”, “galectin-3”, “thioredoxin” and “HMGB-1”, “RNA”, “lung cancer”, “volatile organic compounds”, “electronic nose”, “ion mobility spectrometry”, “GC-MS”, “headspace”, “cell line”, “asbestos”, “exhaled breath”, “breath analysis” and “metabolomics”.

Table 1 summarizes the results from 14 articles describing different biomarkers found in blood and/or pleural effusions. In this table, we only included the data involving the differentiation between individuals who have been diagnosed with MPM and the at-risk group of people exposed to asbestos: healthy asbestos-exposed individuals and/or patients with benign asbestos-related pleura-pulmonary conditions e.g. asbestosis, pleural plaques and fibrosis.

**Table 1:** A summary of the results for diagnostic studies on different biomarkers in blood and/or pleural effusions

Studied groups	Studied marker(s)	Number of patients	ROC-AUC	Threshold	SE	SP	Ref.
MPM vs. healthy AE	SMRP (S)	88 vs. 61	0.806	0.8–1.9 nM	75%–43.2%	68.9%–100%	[15]
	OPN (S)	96 vs. 112	0.724	NA	NA	NA	[23]
	SMPR (S)	96 vs. 112	0.866	NA	NA	NA	[23]
MPM vs. diseased AE	SMRP (S)	74 vs. 28	0.872	0.93nM	80%	82.6%	[19]
	SMRP (PE)	74 vs. 28	0.831	10.4 nM	76.7%	76.2%	[19]
	OPN (P)	96 vs. 33	0.677	NA	NA	NA	[23]
	SMRP (S)	96 vs. 33	0.834	NA	NA	NA	[23]
	SMRP (S)	129 vs. 75	NA	1.6 nM	42%	95%	[25]
	SMRP (S)	129 vs. 75	NA	1.35 nM	53%	88%	[25]
MPM vs. AE (healthy + diseased)	SMRP (S)	117 vs. 86	0.790	1.4–2.5 nM	67%–49%	80%–98%	[28]
	CA125 (S)	117 vs. 86	0.687	6–25 U/ml	52%–9%	80%–98%	[28]
	SMRP+CA125	117 vs. 86	0.801	NA	68%–42%	80%–98%	[28]
	SMRP (S)	85 vs. 212	0.859	2 nM	62%	95%	[30]
	MPF (S)	85 vs. 212	0.847	12.38 ng/ml	68%	95%	[30]
	TRX (S)	57 vs. 34	0.8178	60 ng/ml	71.9%	85%	[40]
	Fibulin-3 (PL)	92 vs. 132	0.99	52.8 ng/ml	71.32%–100%	100%–69.57%	[41]
	SMRP (S)	90 vs. 66	0.810	1.9 nM	60%	89.2%	[17]
	SMRP (S)	31 vs. 204	0.762	0.555–1.56 nM	45.2%–95%	95%–36.8%	[33]
	OPN (PL)	31 vs. 204	0.795	334.5–1423.9 ng/ml	29.5%–95%	95%–31.4%	[33]
	SMRP + OPN	31 vs. 204	0.873	NA	NA	NA	[33]
	SMRP (S)	24 vs. 172	0.725	16.06 nM	64.5%–95%	95%–37.7%	[32]
	OPN (PL)	32 vs. 207	0.780	878.65 ng/ml	62.5%	87.3%	[32]
	Total HMGB1 (S)	22 vs. 20	0.830	15.75 ng/ml	68.8%	84.5%	[42]
	HA HMGB1 (S)	22 vs. 20	1	2 ng/ml	100%–72.73%	5%–100%	[42]
	Fibulin-3 (PL)	22 vs. 20	0.959	NA	NA	NA	[42]
	SMRP (PL)	22 vs. 20	0.934	NA	NA	NA	[42]
	OPN (PL)	22 vs. 20	0.961	NA	NA	NA	[42]
	OPN (S)	76 vs. 69	0.888	48.3 ng/ml	77.6%	85.5%	[35]
	SMRP (S)	24 vs. 92	0.817	1.5 nM	67%	92.5%	[20]
	SMRP (S)	42 vs. 48	0.86	0.62 nM	97.6%	68.9%	[44]
	TRX (S)	42 vs. 48	0.72	156.67 ng/ml	92.9%	77.6%	[44]
	EGFR (S)	42 vs. 48	NA+	19.96 ng/ml	90.5%	64.4%	[44]
	Fibulin-3 (S)	42 vs. 48	NA	51.41 ng/ml	88.1%	66.7%	[44]
	SDC-1 (S)	42 vs. 48	NA	3.77 ng/ml	90.0%	61.9%	[44]
AE vs. healthy non-AE	Fibulin-3 (PL)	136 vs. 43	0.64	21.1 ng/ml	11%–100%	100%–9.30%	[41]
	Total HMGB1(S)	22 vs. 20	0.964	3.05 ng/ml	NA	NA	[42]
	HA HMGB1 (S)	22 vs. 20	0.574	0.45 ng/ml	NA	NA	[42]
“Early-stage” MPM vs. AE (healthy + diseased)	SMRP (S)	12 vs. 66	0.741	2 nM	58%	91%	[17]
	OPN (S)	13 vs. 69	0.906	62.4 ng/ml	84.6%	88.4%	[35]
	SMRP (PE)	74 vs. 63	0.809	11.4–20.8 nM	76.7%–65.1%	69.4%–83.7%	[19]

ROC= Receiver Operating Characteristics curve, AUC = Area under the curve, SE = sensitivity, SP = specificity, MPM = malignant pleural mesothelioma, AE = asbestos-exposed, NA = not available, diseased AE = people with benign asbestos-related conditions (e.g. pleural plaques, asbestosis or pleural effusions), S = serum, PL = plasma, PE = pleural effusions.

#### Protein biomarkers in blood and/or pleural effusions

One of the most extensively evaluated serum biomarkers is soluble mesothelin-related peptide (SMRP), also known as soluble mesothelin. SMRP is a protein derived from the MSLN gene, that is initially translated into a precursor protein of ∼69 kDa. This protein is processed by proteolytic reactions, resulting in a cell-surface bound polypeptide of ∼40 kDa named mesothelin, and a soluble polypeptide of ∼30 kDa named megakaryocyte potentiating factor (MPF). Mesothelin and SMRP have an identical NH_2_-terminus, but a unique COOH-terminus [10]. Mesothelin is expressed on the mesothelium of the pleural, pericardial and peritoneal membrane and plays an important role in cell adhesion and both cell-to-cell recognition and signaling by interaction with Cancer Antigen (CA) 125 [11]. MPF in itself has oncogenic potential by its ability to suppress cell death [12].

Both the diagnostic and prognostic value of SMRP as a potential stand-alone marker have been extensively studied, with reports showing that MPM patients have significantly higher levels of SMRP, which makes this biomarker interesting as a diagnostic tool [13–25]. Asbestos-exposed individuals seem to have higher SMRP concentrations than individuals who haven't been exposed to asbestos, regardless of the presence of pleural disease. Therefore, serum SMRP levels can also be a marker of asbestos exposure [26]. SMRP levels have been studied in both serum (S-SMRP) and pleural effusions (PE-SMRP)[27]. PE-SMRP has a better diagnostic performance in differentiating MPM from other malignancies and asbestos-related benign diseases. Despite of its high specificity, S-SMRP shows a lack of sensitivity. Therefore, further research has focused on the combination of serum mesothelin with several other biomarkers in panels in order to improve their respective diagnostic accuracy. An interesting combination is that of SMRP with CA125. However, combining these biomarkers did not improve sensitivity for detecting MPM over SMRP alone [28]. CA125 is a large transmembrane mucin protein found on the cell surface of mesothelial cells, and is routinely used as tumor marker in ovarian carcinoma. Evidence shows that CA125 is involved in cell-mediated immune response [11].

A correlation has been shown between the serum mesothelin levels and the histological subtype of the tumor. More specifically, patients with epithelioid mesothelioma show higher levels of serum mesothelin than those with sarcomatoid mesothelioma. The same correlation has been observed for MPF [29].

Osteopontin (OPN) and MPF are also biomarkers that show increased levels in patients with established MPM. The diagnostic performance of these markers was evaluated in multiple studies [13, 30–33], but both glycoproteins lack sensitivity as stand-alone biomarkers. OPN is a secreted glycoprotein that facilitates recovery of the organism after injury or infection. It regulates cell migration and stimulates cellular signaling pathways via diverse receptors that can be found on most cell types. OPN also plays an important role in modulating immune and inflammatory responses [34]. It appears that OPN can be useful in the differentiation between asbestos-exposed persons who do not have cancer and mesothelioma patients who have been exposed to asbestos [35]. One study has shown that combining SMRP and OPN improves the diagnostic accuracy over SMRP alone [33]. However, another comparative study did not confirm this result [31]. The same has been observed for the combination of SMRP and MPF [31].

The diagnostic accuracy of SMRP and MPF have been examined and compared with each other in both serum and pleural effusions [36]. Both biomarkers seem to have an equivalent diagnostic accuracy in these biological samples.

Other interesting biomarkers that have been investigated, are C-C chemokine ligand 2 (CCL2) and galectin-3 (LGALS3), both measured in patients with pleural effusions[37, 38]. This restricts their use to a group of patients with an already higher a priori likelihood of mesothelioma than asymptomatic asbestos-exposed individuals. CCL2 is a chemokine involved in the recruitment of mononuclear phagocytes into inflamed and/or neoplastic tissues. LGALS3 is a lectin protein that is abundantly secreted by tumor cells and tumor-associated macrophages. It chemo-attracts macrophages, suppresses T-cell function, and directly supports tumor cell invasion [39]. CCL2 levels follow the same trend as the aforementioned biomarkers and are increased in MPM patients, but unexpectedly, circulating galectin-3 concentrations are decreased in the case of MPM [38]. The diagnostic accuracy of a panel consisting of SMRP, CCL2 and LGALS3 was investigated, and compared with that of SMRP alone [37], with the biomarker combination resulting in a better diagnostic performance. Secretory leukocyte peptidase inhibitor (SLPI), which has versatile tissue-protective functions, has been studied as a potential biomarker in MPM as well, but results showed that this protein does not outperform the abovementioned biomarkers [37].

Cellular pathways involved in redox processes would be expected to produce potential biomarkers in MPM as well. Accordingly, a study showed that thioredoxin-1 (TRX-1), a protein with anti-oxidative activity, is elevated in MPM patients in comparison with asbestos-exposed individuals that did not develop MPM [40].

Fibulin-3 is a member of the extracellular glycoprotein fibulin family and plays an important role in skeletal development [41–43]. Plasma fibulin-3 is able to distinguish MPM patients from controls [41]. SMRP, Fibulin-3 and TRX-1 have also been investigated in a study that also studied the potential of syndecan-1 (SDC-1) and epidermal growth factor receptor (EGFR) [44]. This study showed that SMRP and TRX-1 are the most valuable serum biomarkers for early detection of MPM. SDC-1 is a transmembrane heparan sulphate proteoglycan, which functions as an extracellular matrix receptor and is involved with modulation of neovascularization. EGFR is a member of the receptor tyrosine kinase family that plays an important role in tumorigenesis [44].

HMGB-1, also known as high-mobility group box 1 protein, is found in the nucleus of healthy mesothelial cells, but once these cells are exposed to asbestos, HMGB-1 is translocated to the cytoplasm and into the extracellular space. The release of HMGB-1 induces the secretion of TNF-α by macrophages, resulting in the protection of the asbestos-exposed mesothelial cells against asbestos-related cell death and in chronic inflammatory response [45]. Total serum HMGB-1 levels have been shown to differentiate asbestos-exposed individuals from non-exposed healthy subjects [42]. A specific HMGB-1 isoform, namely hyper-acetylated HMGB-1, even outperforms previously described biomarkers. Hyperacetylation of HMGB-1 translocates this damage-associated molecular pattern to the cytosolic and subsequent extracellular space, promoting inflammation. This marker was able to discriminate between MPM patients and asbestos-exposed individuals without MPM or non-exposed individuals with 100% sensitivity and specificity. Combining fibulin-3 with either total or hyper-acetylated HMGB-1 improved both sensitivity and specificity for differentiating MPM patients from individuals with non-MPM pleural effusions. Nevertheless, there are some limitations that have to be considered with regard to HMGB-1. The sample size in this study was small and the different patient groups were not matched for factors such as age, sex and smoking status, which could lead to confounding effects. In order to use this interesting biomarker in a clinical setting, these results require validation in an independent cohort.

In conclusion, there is a plethora of blood and pleural fluid biomarkers that can potentially be used for early-stage diagnosis of MPM, mostly measured by ELISA-based immuno-enzymatic assays. Nevertheless, most of these components lack sufficient sensitivity and/or specificity in distinguishing MPM patients from healthy asbestos-exposed individuals and persons with asbestos-related benign diseases. In order to clear the way for clinical implementation, certain pitfalls should be taken into account. The study population has to be chosen very carefully as there are confounding variables that influence biomarker levels [46]. For instance, our group described the association of levels of SMRP and MPF with age, glomerular filtration rate (GFR), disease stage and body mass index (BMI) [14].

### Circulating non-coding RNAs

Non-coding RNAs (ncRNAs) are nucleic acids that lack protein-coding potential and contain two major classes: microRNAs (miRNAs) and long non-coding RNAs (lncRNAs).

MiRNA's are small ncRNAs of 17 to 22 nucleotides long, regulating protein translation through several well-characterized mechanisms [47]. MiRNA signatures in tissue and blood have been extensively investigated as diagnostic and prognostic biomarkers in different types of cancers.

Evidence has shown that miRNAs are dysregulated in malignant pleural mesothelioma and that specific miRNAs seem to play a key role in MPM development and progression. Therefore, these miRNAs could be useful as MPM markers [48]. Benjamin *et al*. identified miRNA biomarkers that allow differential diagnosis of MPM [49]. They developed a diagnostic assay that is based on miRNA expression in tissue. This assay is based on hsa-miR-200c and hsa-miR-192 that both show overexpression in lung adenocarcinoma and carcinomas that frequently metastasize to the pleura and on hsa-miR-193-3p that is overexpressed in MPM.

Ak *et al*. found that certain microRNAs in tissue are significantly upregulated in MPM compared to benign asbestos-related pleural effusions [50]. More specifically, the following microRNAs allowed differentiation between malignant and benign disease: hsa-miR-484, hsa-miR-320, hsa-let-7a and hsa-miR-125a-5p.

As miRNAs are in large part packaged within circulating exosomes, they are protected from degradation by circulating enzymes and can be robustly profiled in blood samples. Bononi *et al*. recently showed that several circulating miRNAs in serum, namely miR-197-3p, miR-1281 and miR-32-3p are potential new MPM biomarkers [51]. These miRNAs were upregulated in MPM patients compared to healthy individuals. Intriguingly, upregulated miR-1281 was not only found in MPM patients, but also in non-MPM subjects who had been exposed to asbestos in the past. Based on these findings, further work is necessary to establish the value of circulating miRNAs as reproducible MPM biomarkers.

LncRNAs are non-protein coding RNAs of more than 200 nucleotides long. They play an important role in regulating transcription and there is rising evidence that their aberrant expression plays a role in cancer biology [52], while being very specific for the tissue of origin. Long non-coding RNAs are reported to serve as biomarkers in MPM [53]. Wright *et al*. demonstrated that lncRNA expression tissue profiles allow differentiation between malignant mesothelium and benign pleura [53]. LncRNAs can also be reliably detected in plasma samples, offering the possibility to explore these molecules as biomarkers for MPM.

### Breath analysis: An alternative for blood biomarkers

The search for new and non-invasive biomarkers is currently shifting towards the field of breathomics [54]. Analysis of the exhaled breath of an at-risk population can provide valuable information on the metabolic status of the patient. Breath contains volatile organic compounds (VOCs) arising from endogenous biochemical pathways or from inhaled/absorbed exogenous sources. The concentration of these VOCs usually ranges from the low part per billion (ppb) to the part per trillion (ppt) level. Changes in the VOC-profile reflect changes in processes related to metabolism (host and microbiome-derived), inflammation or tumoral development [54]. Therefore, a selection of VOCs can potentially be used as a diagnostic biomarker to screen for certain diseases such as MPM [55]. To date, researchers have mainly focused on lung cancer within this research field. Nevertheless, there are also a number of studies which investigated the potential of breath analysis as diagnostic tool for pleural mesothelioma.

There are different technologies available that are well suited for breath analysis [56]. The gold standard is gas chromatography-mass spectrometry (GC-MS). This technique allows both identification and quantification of individual compounds with very high sensitivity, and is usually combined with thermal desorption or solid-phase micro extraction (SPME). The downside of this method is its long time-to-result, relative cost and requirement for expert operator staff.

Selected ion flow tube mass spectrometry (SIFT-MS) and proton transfer reaction mass spectrometry (PTR-MS) are both techniques which allow real-time and on-line measurements of VOCs in breath [57, 58]. These methods are both based on chemical ionization of the trace compounds by well-defined reagent ions, resulting in product ions that can be detected and quantified, based on their mass-to-charge ratio (m/z). The sensitivity of SIFT-MS is higher than for PTR-MS, but GC-MS still has the highest sensitivity. Furthermore, PTR-MS and SIFT-MS generate large fragmentation of the compounds in the entire sample at once, limiting their use for unsupervised biomarker detection. Another analytical technique that can be used for breath analysis, is ion mobility spectrometry (IMS). This involves the movement of gas-phase ions that are exposed to an electric field in a drift tube, where they counteract with a drift gas (nitrogen or synthetic air). The product ions gain a constant velocity through the influence of an electrical field and by collision with the drift gas molecules [59]. This velocity depends on the size, mass and shape of the concerning product ions. The advantages of this technique are speed and user-friendliness, allowing low-cost, online sampling.

Recently, sensor technologies based upon pattern recognition like electronic noses (e-noses) have been developed allowing a fast and non-invasive analysis of exhaled breath. These devices are inspired by the mammalian olfactory system and are also known as biomimetic cross-reactive sensor arrays [60]. In contrast to the abovementioned techniques, an e-nose does not allow the identification of individual VOCs as the sensors only recognize a bulk of VOCs giving a breath signature as output. In principle, a standard e-nose has a lower sensitivity and limit of detection compared to other mentioned techniques, which is not an issue as such as long as the technology allows accurate discrimination between certain groups. If, using different methods, cancer-specific VOCs can be defined, then an array of e-nose sensors can be designed that specifically recognizes these cancer-related compounds. With this strategy, the specificity of an e-nose will be higher than that of the standard, more complex technologies.

Dragonieri *et al*. investigated whether an e-nose would allow to distinguish MPM patients from asbestos-exposed individuals without MPM and from healthy controls [61]. They included 13 subjects in each group. Their attempt to separate the breathprints of patients with MPM from those of individuals with similar professional asbestos exposure showed promising results. They were also able to differentiate between the MPM patients and healthy controls based on their breathprint.

The diagnostic potential of these breathprints has been confirmed by Chapman *et al*., who correctly identified patients with MPM, patients with benign asbestos-related diseases and healthy individuals in 88% of cases [62]. De Gennaro *et al*., developed a method based on GC-MS in order to determine discriminatory VOCs among patients with MPM, individuals with long-term occupational exposure to asbestos and healthy controls without asbestos exposure [63]. They demonstrated that cyclohexane and cyclopentane are the dominant VOCs for discriminating between the abovementioned groups.

The potential of IMS for diagnostic purposes was investigated by Cakir *et al*. Discrimination between healthy controls and patients with asbestos-related diseases was possible based on a combination of two VOCs in the IMS chromatogram representing α-pinene and 4-ethyltoluol [64]. Recently, our group published the results of the detection of MPM in exhaled breath by IMS [65]. In contrast to Cakir *et al*., we included MPM patients and were able to discriminate these patients from both healthy non-asbestos exposed individuals as well as asymptomatic asbestos-exposed subjects with a sensitivity and specificity of respectively 87% and 70%.

Despite these promising results, conclusions from these studies cannot be generalized due to the rather limited number of individuals included in each cohort. None of these studies have externally validated their findings, which is a necessary step towards clinical implementation. In addition, further refinements in the reported VOC signatures could lead to substantial increases in diagnostic accuracy. One way to achieve this goal is to investigate which VOCs are originating from the cancer cells themselves rather than from the inflamed stromal environment (the latter being shared between mesothelioma patients and asbestos-exposed individuals without evidence of tumor).

### Searching for mesothelioma cancer cell-specific VOCs

A way to directly home-in on cancer cell-specific VOCs, is to analyze the so-called “headspace” air of *in vitro* cell cultures containing only cancer cells of interest. Different experimental set-ups have been investigated to that end, with most published research focusing on lung cancer. Different methods have been used to analyze the VOCs in the headspace of different cancer cell lines, lung cancer tissue and pleural effusions. The VOCs that have been detected, are very divergent among different reports.

To date, the only study that included a MPM cell line, was performed by Gendron *et al*. They were able to differentiate between different cancer cell lines consisting of adenocarcinoma, squamous cell carcinoma, and mesothelioma using an electronic nose [66]. Distinguishing between the studied cancer cell lines, including a MPM cell line, and normal cells was possible based on the difference in composition of the headspace air over the cells. The degree of discrimination between the different samples was indicated by the Mahalanobis distances (MD). In most cases, the MD between the tumor cell lines and the normal controls was greater than 3 which is the threshold indicating that the e-nose signatures are significantly discriminative. In some cases, the MD was even higher than 5, meaning that the e-nose not just distinguishes the tumor cell lines from other cell types, but also would allow identification of these cell lines. A caveat is that the e-nose platform used in these studies is subjected to drift between sampling sets which jeopardizes reproducibility.

Supplementary Table 1 gives an comparative overview of compounds that were found via both breath analysis (subjects) and headspace analysis (*in vitro* cell cultures). While the only data available relates to lung cancer, it clearly shows that there is some degree of overlap between *in vitro* and *in vivo* detected compounds. Precisely these “shared” VOCs could serve as superior biomarkers for early detection of malignancy. As can be seen in Supplementary Table 1, in headspace of cancerous cell lines it is mainly the concentration of certain aldehydes (acetaldehyde), ketones (2-butanone, cyclohexanon) and alkanes that is significantly decreased or increased compared to the headspace of non-cancerous cell lines or medium only. Conflicting findings have been reported for a few compounds mentioned in Supplementary Table 1. For hexanal, one study involving breath analysis showed an increased concentration for lung cancer, while the *in vitro* results from another study showed a decrease in concentration. As for acetone and 2-butanone, most studies are concordant (i.e. increased concentration in cancer cell lines), but for each of these compounds there is also one study with opposite results.

We plan to perform a similar approach for pleural mesothelioma, i.e. comparing the results from breath analysis with *in vitro* studies on different MPM cell lines in order to see which VOCs are related to aspecific inflammation and which VOCs originate from the cancerous cells themselves.

### Drawbacks involving breath-based biomarkers

As mentioned, VOCs in breath originate from both exogenous and endogenous sources. Nevertheless, only endogenous VOCs can be considered as biomarkers. The fact that VOCs originate from oxidative stress and upregulated metabolism, it could be that it is hard to discriminate between different cancer types. The big challenge in breath testing is to get a better understanding of the biochemical pathways in which cancer-related endogenous VOCs are generated in order to know their origin. Presently, with an exception of acetone and isoprene, little is known about the metabolic processes underlying production of VOC-biomarkers. This shows the importance of *in vitro* experiments, which can provide us with better insights on this matter. When *in vitro* experiments are performed, the cell culture conditions influence the cell culture metabolomics. It will be important for future experiments to closely mimic the physiological conditions in the body instead of working under standard culture conditions. Several studies also showed that the VOC profile can differ among different cell lines of the same cancer [67–70].

Furthermore, studies have shown that the VOC profile in breath shows variability between and within individuals [71, 72]. Although studies are contradictive, it seems that the important factors influencing the breath profile are smoking behavior, body mass index (BMI), gender, age and medication use. Hence it is worthwile to take these into account when studies involving breath testing are performed. However, this is much challenging when it comes to correcting for the contribution of the gut microbiome to the individual VOC spectrum. An additional layer of complexity is generated by the host immune status. Although most available data in that subject pertains to inflammatory/innate immune responses, less is known about the VOCs generated from components of the adaptive immune system. There is one report using *in vitro* experiments revealing that human B-cells also generate a distinct VOC profile [73]. Either way, it is very likely that the immune system may contribute to the VOC profile in breath when the patient is suffering from cancer, infection or other diseases.

Finally, there are also differences in the applied techniques for breath analysis, with their own specific advantages and drawbacks. Furthermore, there is a lack of standardization in analytical technology which makes interpretation of results between different studies difficult.

### Conclusions and future perspectives

The increasing incidence of malignant pleural mesothelioma is not only a problem of the present, but is also a challenge for years to come. Asbestos, the main etiological agent of MPM, is still being processed in developing regions and therefore, its incidence will continue to rise. The early detection of malignancy, including MPM, seems very important in order to improve survival rates. Because current screening tools for MPM generally detect the disease in an advanced stage, there is an ongoing search for new biomarkers that allow the early detection of MPM. As described in this review, many efforts have already been done within this scope, but the search continues as there is still no validated ‘gold standard’. Ideally, potential biomarkers should be non-invasive, robust and easy-to-use. Test-related costs should be minimal and time to analytical result should be sufficiently short.

We aim to develop breath analysis as a point-of care biomarker test that meets these requirements. Breathomics is an increasingly investigated research field showing promising results for early stage diagnosis of MPM. Rigorous studies on large patient cohorts and appropriate controls will determine the clinical validity and utility of breathomics in the diagnosis of mesothelioma. Studies addressing the accuracy in mesothelioma patients versus healthy controls are redundant, as are studies restricted to pleural effusions, as the latter are obtained in patients who have already a high likelihood of MPM.

President Obama introduced the “National Cancer Moonshot” initiative which should accelerate research efforts on prevention, (early) diagnosis and treatment of cancer. Our search for the ‘ideal’ biomarker in malignant pleural mesothelioma fits within the scope of this initiative. Cancer is a disease that affects people in every layer of society and we, scientists, have the obligation to use our knowledge on human health in exploring new ways to improve cancer management. Therefore, future studies should focus on the at-risk population, consisting of people being professionally exposed to asbestos with a latency time of at least 20 years after exposure.

Box 1 **General aspects on biomarker development**

In general, a biomarker gives an indication of the biological state of an organism. More specifically, a diagnostic biomarker should indicate whether a disease is present [74]. In the development of a (diagnostic) biomarker, methodological validation is an important step towards clinical implementation [75, 76]. The aims are to assess the test's reproducibility, repeatability, accuracy and sensitivity/specificity. In the first part of biomarker development, an internal validation is performed. This involves the inclusion of a test set in order to build up a diagnostic model, and subsequent validation of the findings using a validation set. In the next phase, an external validation should be performed, using a separate, prospectively recruited set of test subjects. This essential step yields a better picture of the robustness of the proposed model [77, 78]. Although these validation steps can establish the analytical and clinical validity of the test, it is still essential to ascertain clinical utility: does early detection of disease effectively correlate with better outcome? Can a negative test spare subjects from unnecessary and potentially harmful invasive diagnostic procedures?

## SUPPLEMENTARY TABLE




