# Supplementary material for: Biomarkers for early diagnosis of malignant mesothelioma: Do we need another moonshot?
Source: Oncotarget. 2017 May 17;8(32):53751–62. doi: 10.18632/oncotarget.17910 (PMC5581147; doi:10.18632/oncotarget.17910)
Supplement: Supplementary file 1 [file oncotarget-08-53751-s001.pdf]

Biomarkers for early diagnosis of malignant mesothelioma: Do we need another moonshot?

Supplementary Materials

Supplementary Table 1: An overview of VOCs that were found both through breath analysis and headspace analysis of *in vitro* cell lines in case of lung cancer. See_Supplementary_Table_1.
