# Supplementary material for: Biomarkers for early diagnosis of malignant mesothelioma: Do we need another moonshot?
Source: Oncotarget. 2017 May 17;8(32):53751–62. doi: 10.18632/oncotarget.17910 (PMC5581147; doi:10.18632/oncotarget.17910)
Supplement: Supplementary file 2 [file oncotarget-08-53751-s002.docx]

**Supplementary Table 1:** An overview of VOCs that were found both through breath analysis and headspace analysis of *in vitro* cell lines in case of lung cancer.

| **Class** | **Compound** | **Sample** | **Result** | **Reference** |
| --- | --- | --- | --- | --- |
| **Alkanes** | n-pentane | in vitro  breath  breath  breath | NA  ↑  ↑  NA | (79)  (80)  (81)  (82) |
|  | n-hexane | in vitro  breath  breath | NA  ↑  ↑ | (79)  (83)  (81) |
|  | n-octane | in vitro/breath/ex vivo  in vitro  in vitro | ↑  ↑  NA | (83)  (67)  (79) |
|  | n-decane | breath  in vitro  breath  in vitro | NA  ↑  NA  NA | (84)  (85)  (71)  (79) |
|  | n-undecane | breath  in vitro/breath  breath | NA  NA  NA | (86)  (84)  (71) |
|  | n-nonadecane | in vitro/breath  in vitro | NA  NA | (87)  (85) |
|  | n-eicosane | in vitro/breath  in vitro | NA  NA | (87)  (85) |
| **Alkanes**  **(branched)** | 2-methyl-pentane | breath  in vitro  in vitro | ↑  ↑  NA | (80)  (88)  (79) |
|  | 3-methyl-pentane | breath  in vitro | ↑  NA | (81)  (79) |
|  | 2,4-dimethylheptane | in vitro  breath | ↑  NA | (89)  (71) |
|  | 4-methyl-octane | in vitro  breath | ↑  NA | (89)  (82) |
| **cycloalkanes** | cyclohexane | in vitro  breath | NA  NA | (79)  (71) |
| **Alkenes** | 2-methyl-1,3-butadiene (isoprene) | breath  in vitro/breath  breath  breath | ↓  NA  NA  NA | (86)  (84)  (71)  (90) |
| **Alcohols** | ethanol | breath  in vitro/breath/ex vivo  in vitro  breath  in vitro/breath  in vitro | ↑  ↑  ↑  ↑  ↑  NA | (91)  (83)  (67)  (90)  (92)  (79) |
|  | 1-propanol | in vitro/breath  breath  breath  in vitro | ↑  NA  ↑  NA | (92)  (81)  (90)  (79) |
|  | 2-propanol | in vitro/breath  in vitro | ↑  NA | (92)  (79) |
| **Aldehydes** | pentanal | in vitro  breath  breath | NA  NA  ↑ | (79)  (93)  (90) |
|  | hexanal | HS of blood sample  in vitro  breath  breath  breath  breath  in vitro | NA  ↓  NA  ↑  NA  NA  NA | (94)  (89)  (71)  (95)  (90)  (96)  (79) |
| **Ketones** | acetone | breath  breath  in vitro  breath  in vitro/breath  in vitro | ↓  ↑  ↑  ↑  ↑  NA | (86)  (91)  (67)  (90)  (92)  (79) |
|  | 2-butanone | breath  breath  breath  in vitro  breath  in vitro  breath  breath  breath  in vitro/breath  in vitro | NA  ↑  ↑  ↓  ↑  ↑  ↑  NA  ↑  ↑  NA | (86)  (97)  (91)  (89)  (98)  (70)  (93)  (81)  (99)  (92)  (79) |
|  | 2-pentanone | breath  in vitro  in vitro  breath  breath | ↑  ↑  ↑  NA  ↑ | (91)  (67)  (70)  (93)  (90) |
|  | acetophenone | breath  in vitro  in vitro | NA  ↑  NA | (86)  (70)  (79) |
|  | 2-pentadecanone | in vitro/breath  in vivo | NA  NA | (87)  (85) |
| **Esters** | n-butyl-acetate | breath  in vitro  in vitro  in vitro  in vitro | NA  ↓  ↓  ↓  ↓ | (86)  (67)  (89)  (100)  (88) |
|  | ethylbenzene | breath  breath | NA  ↑ | (101)  (90) |
| **Sulfur compounds** | carbon disulfide | in vitro/breath  (breath) | ↑  (NA) | (92)  (101) |
|  | dimethyl sulfide | in vitro/breath  breath | ↑  ↑ | (92)  (90) |
